# Effect of postpartum family planning intervention and associated factors during child immunization in Addis Ababa, Ethiopia

**DOI:** 10.11604/pamj.2024.47.110.34883

**Published:** 2024-03-06

**Authors:** Sawra Getnet Gelaw, Negussie Deyessa, Achenef Kidane, Ann Evensen, Aschalew Teka, Bethelhem Bokan, Subah Abderehim Yesuf

**Affiliations:** 1 Department of Family Medicine, College of Health Sciences, Addis Ababa University, Addis Ababa, Ethiopia,; 2 Department of Public Health, College of Health Sciences, Addis Ababa University, Addis Ababa, Ethiopia,; 3 Bill and Melinda Gates Foundation, Outbreak Response Consultant, Addis Ababa, Ethiopia,; 4 Department of Family Medicine and Community Health, University of Wisconsin School of Medicine and Public Health, Wisconsin, USA,; 5 World Health Organization, Technical Officer Immunization Program, Addis Ababa, Ethiopia

**Keywords:** Postpartum, family planning, intervention, child immunization, Ethiopia

## Abstract

**Introduction:**

World Health Organization (WHO) recommends postpartum family planning (PPFP) as a critical component of health care that has the potential to save millions of maternal and infant lives in low- and middle-income countries.

**Methods:**

participants in our randomized, controlled trial were mothers coming for vaccination of their child in three selected health centers in Addis Ababa during the first 10 weeks postpartum. Eligible mothers were randomly assigned to intervention (pamphlet-supported counseling about the benefits of family planning) and non-intervention (routine care) arms. Data were collected when mothers came with their infants for a routine measles vaccination at nine months of life. Family planning (FP) use was compared between the groups using logistic regression, and bivariate and multivariate analyses. The study also used Kaplan Meier and Cox regressions to compare the median time of PPFP use and its correlation using SPSS version 26. The research was undertaken from December 2019 to June 2021.

**Results:**

a total of 347 women (177 control, 170 intervention) enrolled in the study. Fifty-eight percent were 24-30 years old. Young age, knowledge about FP, previous use of an FP method, and being married were found to be independent predictors for PPFP use. When comparing intervention and non-intervention groups, there was no significant effect on contraceptive use (adjusted OR 0.633 [95% CI 0.385-1.040]).

**Conclusion:**

pamphlet-supported counseling of mothers in the first 10 weeks postpartum did not increase PPFP at nine months postpartum. Successful interventions will likely require holistic strategies, especially in resource-limited settings. The trial had been registered with clinicaltrials.gov (NCT04521517) on September 24, 2019.

## Introduction

Evidence shows that family planning (FP) is a cost-effective public health intervention with the potential to reduce both maternal and child mortality, WHO defines PPFP as the prevention of unintended pregnancy and closely spaced pregnancies through the first 12 months following childbirth. The general use of properly accepted contraceptives is one of the most cost-effective methods for improving the health of mothers and indirectly their children [1-3]. However, around 222 million women have an unmet need for FP, with the majority of these women living in low- and middle-income countries [4-6]. Globally, there were 213 million pregnancies in 2012, most of which ended in birth. This number will certainly increase as the global population continues to grow, and a large proportion of youth enter their childbearing years, closely spaced pregnancies within the first year postpartum are the riskiest for mother and baby resulting in increased risk for adverse outcomes, such as preterm, low birth weight, and small for gestational age [7]. Citing the prospective definition of unmet need for FP, which uses a postpartum woman´s fertility preferences looking forward at the time of the survey, as many as 50%-90% of women from 17 low- and middle-income countries (LMICs) report an unmet need for postpartum family planning (PPFP), the risk of child mortality is highest for very short (<12 months) birth-to-pregnancy intervals if all couples waited 24 months to conceive again, under-five mortality would decrease by 13%, if couples waited for 36 months, the decrease would be 25% [8].

Women frequently return to fertility and sex before initiating contraception after delivery and do not necessarily understand the risk of pregnancy before the return of menses [9]. For instance, a report on the Demographic and Health Survey (DHS) in Bangladesh data shows that 33% of women resumed sexual activity within three months postpartum, but only 7.2% were using contraception. In a study from Rwanda, these proportions are 73.6% and 1.7%, respectively [10]. The World Health Organization (WHO) recommends PPFP as a critical component of health care that has the potential to meet women´s desire for contraception and save millions of maternal and infant lives in low- and middle-income countries [11].

**Maternal and child health and postpartum family planning:** maternal and neonatal morbidity and mortality are major public health concerns in most developing countries [12]. Family planning can avert more than 30% of maternal deaths and 10% of child mortality if mothers space their pregnancies more than 2 years apart [13]. Closely spaced pregnancies within the first year postpartum are the riskiest for mothers and babies, resulting in increased risks for adverse outcomes, such as preterm, low birth weight, and small for gestational age [14]. Ensuring that every woman has only the number of children she desires is an important means of decreasing maternal mortality. A recent 10-year study of maternal mortality in 46 countries found that the risk of maternal death increases as the number of children per woman rises to four or more. The study also found that maternal deaths declined by 7-35% as the number of children per woman fell [15]. Postpartum family planning, therefore, helps women who have an unmet need to space and limit future pregnancies, while helping to lower rates of maternal and child death, it is a cost-effective way to lower maternal mortality by reducing the number of high-risk births. This is especially critical in Ethiopia where the probability of an adult woman dying from a maternal cause during her reproductive lifespan is about one in 40 [16]. Family planning plays an essential role in improving maternal health, which is one of the Millennium Development Goals (MDGs) [17,18].

**Postpartum family planning in Ethiopia:** since 2002, the Federal Democratic Republic of Ethiopia (FDRE) expanded and promoted the community-based distribution of family planning services through the health extension program [19]. The government has removed all duties and taxes on imported contraceptives, and they are available free of charge at public healthcare facilities [20]. Despite these interventions, the unmet need for contraceptives among postpartum women remains high (86%). Postpartum family planning utilization was low ranging from 10% in Northwest Ethiopia to 38% in Tigray [21]. There are limited intervention studies during the postpartum period in Ethiopia and therefore this study will add to the understanding of potential barriers and facilitators of PPFP uptake. The findings will be useful in modifying practice among the healthcare workers providing care at the maternal, neonatal, and child health (MNCH) clinics.

The objective of the study was to assess the effect of the use of pamphlets supported by counseling during child immunization on the average time of initiation and utilization of Postpartum Family planning in the first 9 months. To assess the sociodemographic characteristics of the mother in predicting the initiation of family planning methods during the postpartum period.

## Methods

**Study design:** the study was a randomized, controlled trial involving mothers coming for their first day, sixth-week, or 10^th^-week vaccination of their infant in the selected health centers. Mothers were randomly allocated into two arms (intervention and non-intervention). Mothers in the intervention arm were thoroughly counseled on the benefits of PPFP method use and also were provided with an FP pregnancy risk assessment toolkit and routine immunization service for their infants. Mothers in the control arm received the routine immunization service for their infants. Baseline and post-intervention data were collected from both groups. The post-intervention data were collected at nine months postpartum when the participants came for routine measles vaccination of their infants. The study period was from February 2020 to March 2021.

**Study setting:** the study was conducted in Addis Ababa, the Federal Democratic Republic of Ethiopia. The city has ten sub-cities and one hundred sixteen woredas. In Addis Ababa, there are a total of 52 hospitals; 12 are governmental and 40 are private hospitals. For each woreda, there is one health center (HC) providing primary health care including curative and preventive services for communities living in the respective woreda. For this study, three HCs were selected from the Tikur Anbesa Hospital (TAH) catchment area if they accepted mothers from any part of Addis Ababa and if they provided all of the following services: 1) the continuum of (antenatal care) ANC, delivery, postnatal care, and child immunization; 2) a selection of at least three modern contraceptive methods, including a barrier method such as a condom, a short-term method such as oral contraceptive pills, and a long-term method such as an implant or intrauterine device; 3) referral services for women wanting permanent methods. Based on these criteria, Teklehimanot, Abinet, and Arada HCs were selected. These HCs were important to the Department of Family Medicine as they were considered teaching health facilities undertaken as community clinical attachments for Family Medicine residents from TAH, Addis Ababa University/College of Health Sciences.

**Study population and sampling strategy:** the study included all mothers in the reproductive age group who gave birth to a live child and who came for their first day, sixth week, or tenth week of child vaccination services, with the inclusion and exclusion criteria listed below.

**Inclusion and exclusion criteria:** all healthy women above the age of 18 who were attending their child´s first day, sixth week, and tenth week vaccination, were willing to continue child vaccination in the HC, and were willing to participate in the study were eligible for recruitment. Women needed to be able to consent to the study. Women who already started contraception by the recruitment date including those who had sterilization via hysterectomy, bilateral tubal ligation, and/or bilateral oophorectomy were excluded. Women who were not the biological mother of the index child for vaccination, those who were not fit to respond adequately to questions (including those who were sick and/or suffering from an acute medical condition), or mothers who did not have either a personal or home phone were excluded from the study.

**Intervention:** in the study, all mothers coming for child immunization on their first-day, sixth-week, and tenth-week program had a baseline interview about FP and were then randomly assigned to either the intervention or non-intervention group. Randomization to control and intervention arms was at the level of the HC. Participants allocated to the experimental group received the intervention package (described below), while those allocated to the control group received usual care (child immunizations as per routine schedule). The intervention package included a pamphlet describing PPFP including methods and their timing, advantages, and disadvantages. The pamphlet was supported by one-on-one FP counseling by trained personnel using a checklist. They were also taught how to use the pamphlet. To avoid communication between mothers, counseling was undertaken just before discharge from the clinic (after the children were vaccinated and baseline data was collected). A trained nurse was assigned to counsel women in the intervention group about FP. The healthcare workers in the immunization room and the data collector were blinded to the intervention.

Data were collected with a structured interview by trained nurses. The baseline data collection was at the initiation of the study. The final data collection was at nine months postpartum when the mothers came to the health facility for measles vaccination of their child. If mothers did not come for the measles immunization as scheduled, they were contacted using their phones or health extension workers to assess the use of modern family planning methods.

**Sample size determination:** the sample size for the study was determined using the formula for estimation of proportion in two populations with the assumption of 95% confidence level, power 80%, and by assuming an expected prevalence of PPFP in Addis Ababa of 39% [22]. Estimating our intervention would improve use by a minimum of 15%, a total of 173 women in each group would be required. To compensate for non-response or loss to follow-up, an additional 10% of the sample was to be included for a total of 190 mothers for the intervention and 190 mothers for the control group (total of 380 respondents).

**Sampling procedure:** once an eligible mother had the baseline interview, she was assigned either to the intervention or non-intervention group based on two or four blocks randomly generated by Office Excel, printed on cards, and put in a sealed envelope for the mothers to choose at random.

**Data collection:** all eligible mothers who were willing to participate were interviewed at baseline. A pre-tested, structured, interviewer-administered questionnaire was used to collect data from study participants by trained personnel. The questionnaire had sociodemographic, reproductive health, and other conditions related to FP utilization. The questionnaire was developed by adopting standard questions related to reproductive health including FP. The questionnaire was pre-tested in a non-study HC in Addis Ababa, and amendments were made to improve the questionnaire. Two nurses and Family Medicine residents were trained for two days on how to administer the questionnaire and on ethical issues including privacy and confidentiality. The enumerators also participated in the pre-testing of the questionnaire and were not blinded to the women´s intervention status. A professional trained in clinical trials supervised and monitored the data collection and the randomization process. The Principal Investigator, assisted by AAU faculty, actively supervised the data collection for consistency and completeness. The Principal Investigator was blinded to the allocation of mothers and during data collection. The completed questionnaires were checked daily for completeness, and then coded. The data was compiled in an Office Excel database.

**Dependent variable:** the primary outcome variables were the overall utilization of family planning by the ninth month, postpartum family planning, and the time to use. Independent variables: Socio-demographic characteristics age, marital status, educational status (mother´s/spouse´s), occupation, religion. Family planning and reproductive history parity, previous FP history, place of delivery, knowledge of FP methods, and breastfeeding practice.

**Data analysis:** the data was analyzed using SPSS Version 26. Descriptive and analytical statistics were computed. Description of sociodemographic information and knowledge, attitudes, and practices regarding reproductive health and FP were made at baseline. Intention to treat analysis was done and a comparison was made in the proportion of FP utilization during the postpartum period between intervention and non-intervention groups using bivariate analysis, followed by multivariable analysis using logistic regression (after adjustment for possible confounders). The study assessed the median time to the first date of FP utilization between intervention and non-intervention using Kaplan-Meier analysis. We used Cox regression analysis to assess for a hazard ratio of utilizing FP between intervention and non-intervention groups crudely and after adjusting for possible confounders. For the multivariable analysis, sociodemographic information and conditions related to reproductive health and FP that are associated (including borderline) with both the outcome and the intervention status were considered as possible confounders and included in the models. To assess for possible effect modification and mediation, step-by-step inclusion of variables was made. Conditions that had an association with a p-value less than 0.05 were considered as having an association with PPFP. To assess predicting factors related to the utilization of PPFP, variables related to the outcome variables in the model with higher R^2^were included.

**Ethical considerations:** ethical approval was received from the Institutional Review Board (IRB) office, College of Health Sciences, Addis Ababa University. Permission to collect data was sought from the respective HCs. Participants were informed about the aim of the study, the procedures of data collection, and the required activities. Written informed consent was obtained for inclusion in the study. Participants were informed that participation in the study was fully voluntary and that they could withdraw from the study at any time and/or decline to answer any of the questions. They were also informed that non-participation in the study would have no impact on the immunization of the child or any service given in the HC. Privacy would be maintained during data collection as well as during the intervention. Confidentiality of information was maintained by removing individual identifiers. Written consent was used for willing participants.

## Results

A total of 347 women were enrolled in the study (170 in the intervention group and 177 in the non-intervention group). More than half of the participants were 24 -30 years old, most mothers Exclusively Breast Feeding, more than half of mothers knew about Family Planning methods, and a similar proportion in both groups (Table 1, Table 2, Table 3). Immediate postpartum FP use was low at baseline 73 (21%) followed by an increase in use in the first 2 months of the postpartum period to 290 (82.6%). There was no statistically significant change in overall contraceptive use (crude OR 0.658 [95% CI 0.418-1.037], adjusted OR 0.633 [95% CI 0.385-1.040]) nor in the timing of use after two counseling sessions on average at 9 months postpartum. Age, previous contraceptive use, knowledge about FP, previous discussion of FP with their partner, and being married were found to be independent predictors for FP use during the postpartum period. On binary logistic regression, being married was associated with four times higher FP use in the intervention group as compared to the non-intervention group (crude OR 4.388 [95% CI 1.801 - 10.689, P=0.001]) (Table 4).

**Table 1:** sociodemographic status of mothers by intervention status

Variable name	Intervention; n(%)	Non-intervention; n(%)	X^2^	P-value
**Age group (years)**			2.123	0.346
18-23	30 (57.7)	22 (42.3)		
24-30	97 (48.5)	103 (51.5)		
31-49	43 (45.3)	52 (54.7)		
**Religion**			0.168	0.682
Christian	130 (49.6)	132 (50.4)		
Muslim	40 (47.1)	45 (52.9)		
**Marital status**			1.990	0.158
Not married	8 (34.8)	15 (65.2)		
Currently married	162 (50)	162 (50)		
**Educational status**			2.398	0.494
Not educated	17 (54.8)	14 (45.2)		
Primary	73 (46.8)	83 (53.2)		
Secondary	63 (52.9)	56 (47.1)		
Diploma and more	17 (41.5)	24 (58.5)		
**Spouse educational status**			2.039	0.564
Not educated	4 (66.7)	2 (33.3)		
Primary	34 (43.6)	44 (56.4)		
Secondary	77 (51.7)	73 (48.7)		
Diploma and more	47 (50.5)	46 (49.5)		

**Table 2:** women’s reproductive history by intervention status

Variable name	Intervention; n(%)	Non-intervention; n(%)	X^2^	P-value
**Parity**			0.425	0.515
1-2	116 (49.8)	117 (50.2)		
3 and more	81 (53.5)	53 (46.5)		
**Fertility intention**			0.208	0.648
1-2	34 (48.6)	36 (51.4)		
3 and more	143 (51.6)	134 (48.4)		
**Postpartum follow up**			0.544	0.461
Yes	126 (49.8)	127 (50.2)		
No	51 (54.3)	43 (45.7)		
**Exclusive breastfeeding**			1.067	0.587
Yes	140 (51.1)	134 (48.9)		
No	37 (50.7)	36 (49.3)		
**Pregnancy interval**			0.039	0.981
<24 months	18 (48.6)	19 (51.4)		
24-47 months	39 (48.8)	41 (51.2)		
48 or more months	60 (50)	60 (50)		

**Table 3:** mothers’ Family Planning history by intervention status

Variable name	Intervention; n(%)	Non-intervention; n(%)	X^2^	P-value
**Knows about family planning (FP)**			0.327	0.567
Yes	169 (51.4)	160 (48.6)		
No	8 (44.4)	10 (55.6)		
**Ever used an FP method**			0.347	0.556
Yes	142 (51.8)	132 (48.2)		
No	35 (47.9)	38 (52.1)		
**Wants to limit/space birth**			0.975	0.323
Yes	173 (51,5)	163 (48.5)		
No	4 (36.4)	7 (63.6)		
**Ever been counseled during your last pregnancy**			0.084	0.773
Yes	133 (50.6)	130 (49.4)		
No	44 (52.4)	40 (47.6)		
**Ever discussed with the partner**			0.083	0.773
Yes	149 (50.7)	145 (49.3)		
No	28 (52.8)	25 (47.2)		

**Table 4:** family planning variables by intention to treat

Variable name	Intervention; n(%)	Non-intervention; n(%)	Crude OR	95% CI	P-value
**Knows about family planning (FP)**				2.65 - 25.69	0.000
No	8 (44.4)	10 (55.6)	1		
Yes	169 (51.4)	160 (48.6)	8.250		
**Ever used a FP method**				2.14 - 6.26	0.000
No	35 (47.9)	38 (52.1)	1		
Yes	142 (51.8)	132 (48.2)	3.663		
**Wants to limit/space birth**				2.90 - 181.38	0.003
No	4 (36.4)	7 (63.6)	1		
Yes	173 (51.5)	163 (48.5)	22.941		
**Ever been counseled during your last pregnancy**			1	0.97 - 2.68	0.066
No	44 (52.4)	40 (47.6)			
Yes	133 (50.6)	130 (49.4)	1.613		
**Ever discussed with the partner**				1.54 - 5.08	0.001
No	28 (52.8)	25 (47.2)	1		
Yes	149 (50.7)	145 (49.3)	2.800		

## Discussion

This study was done to evaluate the effect of pamphlet-supported FP counseling in the postpartum period during child immunization on FP use and timing of use. There was an overall increase in FP use in both groups during the first two months postpartum., Most mothers started to use a method before two months postpartum. Unlike a study done in the northern Tigray region of Ethiopia where contraception use was very low, this study demonstrated contacts or visits for vaccination during the first year of an infant´s life offer multiple opportunities for FP counseling and services for women [23].

There was no significant difference in FP use or timing of use between intervention and non-intervention groups. The same findings were seen in studies done in the resource-limited settings of Ghana, Zambia, and the Democratic Republic of Congo evaluating the integration of FP messages into child immunization services. Some of the non-modifiable limitations were also part of the current study which is the setting in addition to the impact of COVID-19 on our study where there were vaccination campaigns undertaken through outreach activities which could compromise the quality of counseling and family planning service provision (more than half of the staff were on outreach activities and the rest were allocated to cover multiple service units. e.g. A nurse was supposed to work at ANC as well as Family Planning units at the same time [24-26].

**Strengths and limitations:** the study employed a randomized design, and we achieved a matching of pertinent baseline characteristics and important covariates between the two groups during the pre-intervention period. However, an important limitation was that the study was conducted in a health facility where there was limited space. Nursing mothers did not want to wait to be seen in two separate rooms (one for data collection and another for counseling). Hence, blinding the data collectors wasn't possible because of the lack of additional private space to counsel mothers. On average, mothers could get a minimum of two counseling sessions. It could have been more effective if the counseling had been delivered at each postpartum visit. The study was carried out in lower-level facilities; findings may not be generalizable to higher-level health facilities. Most importantly the intervention was undertaken during the COVID-19 era when almost all health facility staff were mostly assigned to provide outreach for COVID-19 vaccination campaign activities, and all facilities were trying to continue providing the usual health care service with a limited number of human resources.

## Conclusion

This study revealed a significant overall increment in contraceptive use during the first two months postpartum. Child immunization visits should be considered as a key intervention point in MCH care. Pamphlet-supported counseling of mothers in the first ten weeks postpartum did not increase PPFP at 12 months postpartum. Successful interventions will likely require holistic strategies, especially in resource-limited settings.

### What is known about this topic

- *There is a high unmet need in PPFP, especially in resource-limited settings;*
- *Child immunization is considered a high-impact practice (HIP) opportunity in meeting PPFP unmet needs*.

### What this study adds

- *Strengthens the evidence to contextualize strategies in PPFP interventions, especially in resource-limited settings;*
- *Understanding the impact of the COVID-19 pandemic and also vaccination campaigns help guide PPFP implementation strategies*.
